# Correction: Is no test better than a bad test: Impact of diagnostic uncertainty on the spread of COVID-19

**DOI:** 10.1371/journal.pone.0247129

**Published:** 2021-02-10

**Authors:** Nicholas Gray, Dominic Calleja, Alexander Wimbush, Enrique Miralles-Dolz, Ander Gray, Marco De Angelis, Elfriede Derrer-Merk, Bright Uchenna Oparaji, Vladimir Stepanov, Louis Clearkin, Scott Ferson

The title of this article is incorrect. The correct title is: Is no test better than a bad test: Impact of diagnostic uncertainty on the spread of COVID-19. The correct citation is: Gray N, Calleja D, Wimbush A, Miralles-Dolz E, Gray A, De Angelis M, et al. (2020) Is no test better than a bad test: Impact of diagnostic uncertainty on the spread of COVID-19. PLoS ONE 15(10): e0240775. https://doi.org/10.1371/journal.pone.0240775

## References

[1] Gray N, Calleja D, Wimbush A, Miralles-Dolz E, Gray A, De Angelis M, et al (2020) Is “no test is better than a bad test”? Impact of diagnostic uncertainty in mass testing on the spread of COVID-19. PLoS ONE 15(10): e0240775 10.1371/journal.pone.0240775 33085693 PMC7577497

